# TLR22-Induced Pro-Apoptotic mtROS Abets UPR^mt^-Mediated Mitochondrial Fission in *Aeromonas hydrophila*-Infected Headkidney Macrophages of *Clarias gariepinus*

**DOI:** 10.3389/fimmu.2022.931021

**Published:** 2022-07-04

**Authors:** Manmohan Kumar, Shagun Sharma, Munira Haque, Jai Kumar, Umesh Prasad Sah Hathi, Shibnath Mazumder

**Affiliations:** ^1^Immunobiology Laboratory, Department of Zoology, University of Delhi, Delhi, India; ^2^Faculty of Life Sciences and Biotechnology, South Asian University, New Delhi, Delhi, India

**Keywords:** *A. hydrophila*, TLR22, mtROS, UPR^mt^, mitochondrial fission, apoptosis

## Abstract

Toll-like receptors (TLRs) are epitomized as the first line of defense against pathogens. Amongst TLRs, TLR22 is expressed in non-mammalian aquatic vertebrates, including fish. Using headkidney macrophages (HKM) of *Clarias gariepinus*, we reported the pro-apoptotic and microbicidal role of TLR22 in *Aeromonas hydrophila* infection. Mitochondria act as a central scaffold in the innate immune system. However, the precise molecular mechanisms underlying TLR22 signaling and mitochondrial involvement in *A. hydrophila*-pathogenesis remain unexplored in fish. The aim of the present study was to investigate the nexus between TLR22 and mitochondria in pro-apoptotic immune signaling circuitry in *A. hydrophila*-infected HKM. We report that TLR22-induced mitochondrial-Ca^2+^ [Ca^2+^]_mt_ surge is imperative for mtROS production in *A. hydrophila*-infected HKM. Mitigating mtROS production enhanced intracellular bacterial replication implicating its anti-microbial role in *A. hydrophila*-pathogenesis. Enhanced mtROS triggers *hif1a* expression leading to prolonged *chop* expression. CHOP prompts mitochondrial unfolded protein response (UPR^mt^) leading to the enhanced expression of mitochondrial fission marker *dnml1*, implicating mitochondrial fission in *A. hydrophila* pathogenesis. Inhibition of mitochondrial fission reduced HKM apoptosis and increased the bacterial burden. Additionally, TLR22-mediated alterations in mitochondrial architecture impair mitochondrial function (ΔΨ_m_ loss and cytosolic accumulation of cyt *c*), which in turn activates caspase-9/caspase-3 axis in *A. hydrophila*-infected HKM. Based on these findings we conclude that TLR22 prompts mtROS generation, which activates the HIF-1α/CHOP signalosome triggering UPR^mt^-induced mitochondrial fragmentation culminating in caspase-9/-3-mediated HKM apoptosis and bacterial clearance.

## Introduction

Oxidative burst is the rapid release of reactive oxygen species (ROS), which plays a crucial role in intracellular redox profile influencing a wide variety of signaling pathways (1). It also represents one of the most proficient defense arsenals in host innate immunity against pathogens (2). Macrophages release copious amounts of ROS and the two well-recognized sources are NADPH oxidase (NOX) and mitochondria (3). The production of mitochondrial ROS (mtROS) is primarily attributed to the oxidation of electron transport chain (ETC) metabolic intermediates (3), and the complex I, complex III of the mitochondrial ETC serve as the major sites for mtROS production (4). mtROS were initially thought of as an unwanted adjunct of oxidative metabolism however, recent studies have suggested that macrophages exploit mtROS as the unswerving antimicrobial agent to combat pathogens (5, 6), implicating mitochondria act as a central hub in innate immunity. Though the role of mtROS in anti-microbial defense has been reported in fish (7) nonetheless the underlying molecular mechanism that activates the process in fish remains nebulous.

Toll-like receptors (TLRs) represent an evolutionarily conserved family of pattern-recognition receptors (PRRs) and represent a cornerstone of the fish innate immune response (8). TLRs recognize pathogens *via* pathogen-associated molecular patterns (PAMPs) and endogenous danger signals *via* damage-associated molecular patterns (DAMPs) released by damaged or dying cells (9). Amongst the TLR repertoire, TLR22 has been reported in non-mammalian aquatic animals including fish (10). The role of TLR22 in fish immunity is not well understood and the presence of this receptor in immune and non-immune tissues suggests it to be a multifaceted molecule (11). Previous studies have implicated the role of TLR22 in the fish immune response against microbial infection (12–15). Recently, we reported the anti-bacterial and pro-apoptotic role of TLR22 in fish macrophages (16) but how it contrives innate immune signaling pathways in fish needs to be investigated. Although TLR signaling has been implicated in mtROS production (5), but the primal role of TLR22 has not been reported.

Bacterial infections lead to surfeit consumption of cellular oxygen triggering hypoxia (17). The hypoxia-inducible factor-1 (HIF-1) plays an integral role in the body’s response to hypoxia. It consists of an inducible α subunit (HIF-1α/-2α) and a constitutive β subunit (HIF-1β). HIF-1α is expressed virtually in all innate and adaptive immune cells while the expression of HIF-2α is limited to endothelial cells and certain immune cells (17). Under hypoxic conditions, HIF-1α/-2α dimerizes with HIF-1β and binds with the hypoxia response elements (HREs) in the nucleus (18), initiating the transcription of genes involved in tissue homeostasis and immune response (19). mtROS has been reported as one of the key contributing factors in propagating hypoxia (20) but the exact mechanisms remain poorly defined. Hypoxia has been reported to exert host protective effects in fish by aiding the production of pro-inflammatory cytokines and nitric oxide vital for controlling bacterial burden (21). However, the exact immune signaling mechanisms regulated by HIFs in fish remains elusive.

The fitness of mitochondria is of paramount importance for cellular health and metabolism. The organelle remains dynamically interconnected, undergoing incessant cycles of fission and fusion which is essential for mitochondrial quality control (22). Mitochondrial fission helps in confiscating damaged mitochondria and is triggered by several factors of which dynamin-related protein 1 (Drp1) is important (23). Recent evidence though suggests mtROS is interlinked with mitochondrial fission and fusion (23, 24) but the intricate mechanisms remain unclear. Additionally, there are also reports implicating the importance of mitochondrial dynamics in regulating the outcome of immune response (25) but its role in fish immunity remains elusive.

Unfolded protein response (UPR) is employed to overcome cellular stress and restore proteostasis (26). Pathogenic assault leads to the aggregation of unfolded or misfolded proteins in mitochondria triggering mitochondrial UPR (UPR^mt^) (27–29). UPR^mt^ triggers the induction of mitochondrial chaperones like Hsp60 to maintain protein homeostasis in the organelle (30). Additionally, it also induces certain genes for mitochondrial biogenesis, mitochondrial fission, and the repair and recovery of damaged mitochondria (30).

*Aeromonas hydrophila*, a Gram-negative bacterium is responsible for fatal hemorrhagic septicemia, enteritis, red body disease, and motile Aeromonas septicaemia [MAS] in fish (31). In mammals including humans, it is associated with gastroenteritis, septicaemia, wound infections, and extra-intestinal infections (32). The virulence of *A. hydrophila* has been attributed to its diverse range of virulence factors which poses a difficulty in understanding its pathogenesis. Previous studies have documented *A. hydrophila* induces apoptosis of fish macrophages, involving extrinsic and intrinsic caspases (33). Nevertheless, the signaling mechanism triggering apoptosis in *A. hydrophila*-infected cells needs to be investigated.

It is important to note that information on the physiological processes of mitochondria in the innate immune system had been limited to the mammalian system only. To the best of our knowledge, no direct evidence has been yet presented regarding the role of TLR22 and mitochondrial processes in the fish immune system. In fish, headkidney (HK) is a primary immunocompetent organ and serves as a rich source of macrophages (34). In this study, we have studied the role of TLR22 in triggering the mitochondrial response and shaping the immune outcome in *A. hydrophila* pathogenesis in fish.

## Materials and Methods

### Animal Care and Maintenance

Catfish (*Clarias gariepinus*, 120-150 g; 28 ± 2.5 cm) were procured locally and maintained in 50 L tanks under natural photoperiod. The studies were carried out according to the guidelines issued by Committee for the Purpose of Control and Supervision of Experiments on Animals (CPCSEA), Govt. of India and permitted by Animal Ethics Committee (DU/ZOOL/IAEC-R/2013/33), University of Delhi. Fish were acclimatized for 15 days prior to the experiments and fed with chicken liver *ad libitum* (33).

### HKM Isolation, Infection and Inhibitor Studies

The fish were euthanized using MS 222 (Sigma), headkidney excised aseptically and HKM were isolated using 34/51% percoll gradient as described earlier (33). The HKM were infected with *A. hydrophila* (MOI 1:50) for 1 h and extracellular bacteria was removed using chloramphenicol (30 µg/mL) as described earlier (16).

The HKM were pre-incubated separately with mPTP inhibitor [Cyclosporin A (CsA), 5 µM, Sigma], mitochondrial Ca^2+^ uniporter (MCU) inhibitor [Ruthenium Red (RR), 20 µM, Sigma], caspase-9 inhibitor [Z-LEHD-FMK, 7.5 µM, Sigma], DRP-1 inhibitor [Mdivi-1, 25 µM, Sigma], HIF-1α inhibitor [Dimethyl-bisphenol A (di-BPA), 200 µM, Abcam], mtROS inhibitor [YCG063, 10 µM, Calbiochem], caspase-9 inhibitor [Z-LEHD-FMK, 7.5 µM, Biovision], caspase-3 inhibitor [Ac-DEVD-CHO, 10 µM, Sigma], for 1 h and then infected with *A. hydrophila* as mentioned earlier (16). The inhibitor concentrations used in the study had no adverse effects on the viability of HKM and bacterial growth *per se* (data not shown).

### siRNA Transfection

*tlr22* and *chop* gene was knocked out using specific siRNA (**Table 1**) using HiPerFect Transfection Reagent (Qiagen) as per manufacturer’s instructions. HKM were transfected with sc-siRNA or specific-siRNA-HiPerFect complex and the HKM were incubated at 30°C for 16 h and then infected with *A. hydrophila* as described earlier (33). The knockdown of both the genes was confirmed by RT-qPCR (**Figure S1**).

**Table 1:** List of siRNAs.

S. No.	Gene	siRNA sequence
1.	*tlr22*	Sense: 5’-CCUUUAUCUCUGAGAGGUA-3’ Antisense: 5’-UACCUCUCAGAGAUAAAGG-3’
2.	*chop*	Sense: 5’-AUGAAGACUUGCAAGAUAU-3’ Antisense: 5’-AUAUCUUGCAAGUCUUCAU-3’

### Cloning and Sequencing of *dnm1l* gene

Degenerate primers were designed using *dnm1l* homologous sequences of fishes available in the NCBI database. The cDNA was amplified using degenerate primers and the amplified product was eluted using QIA quick gel extraction kit (Qiagen). The amplified product was cloned into pGEM-T EASY vector (Promega) and sequenced (Macrogen). The sequence obtained (**Supplementary Table 1**) was aligned to nBLAST and submitted to the NCBI database (Accession no. MZ882392).

### RT-qPCR

HKM (1 × 10^7^) pre-incubated with inhibitors or transfected with specific siRNA were infected with *A. hydrophila* and the cultures were terminated at indicated time points. The total RNA was isolated using TRI reagent (MRC) as per the manufacturer’s instructions. The cDNA was prepared using Revert Aid First Strand cDNA synthesis (Thermo Fischer Scientific) from 1 µg of DNase-treated RNA as described earlier (16).

Gene expression studies were performed using ViiA Real-Time PCR system (ABI) and SYBR green PCR Master Mix (ABI) with specific primers (**Table 2**). The expression of the genes was quantitated by comparative ΔΔC_T_ method and normalized against *β-actin* (housekeeping gene) as described earlier (16).

**Table 2:** List of RT-qPCR primers.

S.No.	Gene name	Primer sequence	Product size (bp)	Accession number
1.	*hif1a*	F: 5’-TGACCTTGAGATGCTCGCTC-3’ R: 5’-AAGTGCTGGATGTTGGCGA-3’	161	KC011345.1
2.	*chop*	F:5’-GTTGGAGGCGTGGTATGAAG-3’ R: 5’-GAAACTCCGGCTCTTTCTCG-3’	104	LK054407.1
3.	*hspd1*	F: 5’-GGTTCTCATGGAAAAGCAGCA-3’ R: 5’-GGCAGATTTCAACCCTTGTGT-3’	132	KT368136.1
4.	*dnm1l*	F: 5’-GAGTCTGGTTGGCAGAGACC-3’ R: 5’-CACTCGTCTTTCTCCGGTCC-3’	110	MZ882392
4.	*actb* *(β-actin)*	F: 5’-CTCCCCTGAACCCTAAAGCC-3’ R: 5’-TCAGTTCAGAGATGAAGCCTGG-3’	167	KJ722166.1

### Assessment of Mitochondrial Ca^2+^ [(Ca^2+^)_mt_] Levels

The (Ca^2+^)_mt_ levels were measured using Rhod-2/AM (Molecular Probes). HKM (2 × 10^6^) were pre-incubated with Ruthenium Red or transfected with *tlr22*-siRNA and then infected as mentioned earlier. The cells were washed at 1 h p.i. and stained with Rhod-2/AM (50 nM). The excess dye was washed to remove any unbound dye then resuspended in 1× PBS and the changes in fluorescence intensity levels were measured at Ex_552_ and Em_581_ using microplate reader (BMG Labtech).

### Measurement of Mitochondrial ROS (mtROS) Production

The mtROS levels were determined using MitoSOX™ Red mitochondrial superoxide indicator (Molecular Probes). HKM (2 × 10^6^) were pre-incubated with YCG063, RR or transfected with *tlr22*-siRNA and then infected with *A. hydrophila* as described above. At 4 h p.i., the HKM were washed and incubated with MitoSOX (5 µM) at 30 °C for 20 min in dark. The excess dye was washed and the changes in fluorescence intensity levels were measured at Ex_510nm_ and Em_580nm_ using microplate reader (BMG Labtech).

In a parallel study, fluorescence microscopic analysis was also done for monitoring the changes in mtROS levels. For this, HKM were pre-incubated with YCG063, RR or transfected with *tlr22*-siRNA, infected with *A. hydrophila*, and then incubated with MitoSOX described above. The nucleus of the cells was stained with DAPI (100 µg/mL, Sigma) for 15 min at 30 °C in dark. The HKM were washed, mounted and observed under fluorescence microscope (×40, Zeiss Imager, Z2).

### Apoptosis Assays

#### Hoechst 33342 Staining

HKM (1 × 10^6^) were pre-incubated with YCG063, CsA, di-BPA, Mdivi-1, Z-LEHD-FMK, Ac-DEVD-CHO or transfected with tlr22-siRNA, *chop*-siRNA and infected with *A. hydrophila* as described above. At 24 h p.i., HKM were washed, stained with Hoechst 33342 as described earlier (14) and the slides were visualized under fluorescence microscope (×40, Zeiss Imager, Z2). Hoechst-positive and Hoechst-negative HKM were enumerated and the graph was plotted as % Hoechst-positive HKM.

#### Caspase Assays

Caspase-9 activity (LEHDase) and caspase-3 activity (DEVDase) were monitored using colorimetric caspase-9 and caspase-3 assay kit (Biovision) respectively following the manufacturer’s instructions and using reagents provided with the assay kits. HKM (1 × 10^6^) pre-incubated with CsA, Mdivi-1, Z-LEHD-FMK, Ac-DEVD-CHO or transfected with *tlr22*-siRNA were infected with *A. hydrophila* as mentioned earlier. The HKM were collected at 24 h p.i., washed, re-suspended in chilled lysis buffer (50 μL) and incubated at 4°C for 1 min. Following incubation, the cell lysate was centrifuged at 10, 000 × g for 5 min at 4°C. Supernatant (50 μL) was mixed with 2× reaction buffer supplemented with DTT (10 mM), PMSF (5 mM). Then, 5 μL of substrate (LEHD-pNA for caspase-9 and DEVD-pNA for caspase-3) was added and incubated at 30°C for 5 h. The absorbance was read at A_405nm_ (Epoch2, BioTek) and the relative fold change in the activity of caspase-9 and caspase-3 were calculated.

### Confocal Microscopy

The mitochondrial morphology was observed using MitoTracker Green (Molecular Probes). HKM (1 × 10^6^) were infected with *A. hydrophila* for 24 h p.i., washed and then loaded with MitoTracker Green (50 nM) for 30 min at 30 °C. The nucleus of the HKM was stained with DAPI (1 µg/mL) for 15 min at 30 °C. Excess dye was removed by washing, slide mounted and visualized under fluorescence microscope (×100, Nikon Eclipse Ti2).

### Measurement of Cytochrome *c* (Cyt *c*) Release

The Cyt *c* release was studied according to the reported method (35). HKM (1 × 10^6^) pre-incubated with CsA, Mdivi-1 or transfected with *tlr22*-siRNA were infected with *A. hydrophila* as described above. The HKM were washed at 24 h p.i., and homogenized in buffer A (50 mM Tris, 1 mM PMSF, 2 mM EDTA, pH 7.5), followed by addition of 2% glucose to remove the impurities and centrifuged at 2,000 × g for 10 min at 4°C. The supernatant was collected to detect the release of Cyt *c* in cytoplasm and the pellet was re-suspended in buffer B (50 mM Tris, 2 mM EDTA, pH 5) to obtain mitochondrial fraction. The mixture was centrifuged at 5,000 × g for 30 s at 4°C and pellet was re-suspended in TE buffer. The supernatant and pellet were treated with ascorbic acid (500 mg/mL) for 5 min and absorbance was read at A_550nm_ (Epoch2, BioTek).

### Determination of Mitochondrial Membrane Potential (Δψ_m_)

The Δψ_m_ was studied using Rhodamine 123 (Molecular Probes). HKM (1 × 10^6^) pre-incubated with CsA, Mdivi-1 or transfected with *tlr22*-siRNA were infected with *A. hydrophila* as described above. At 24 h p.i., HKM were washed and incubated with Rhodamine 123 (10 µM) for 30 min at 37°C in dark. The unbound dye was removed by washing and changes in fluorescence intensity were measured at Ex_511nm_ and Em_534nm_ (BMG Labtech).

### Statistical Analysis

Statistical analysis was performed with IBM SPSS 25. For comparison, one-way ANOVA with Bonferroni *post-hoc* test was used to compare the means between the groups. *p < 0.05 was considered as level of statistical significance.

## Results

### TLR22-Induced (Ca^2+^)_mt_ Flux Prompts Antimicrobial mtROS Generation in *A. hydrophila*-Infected HKM

**Figure 1:**
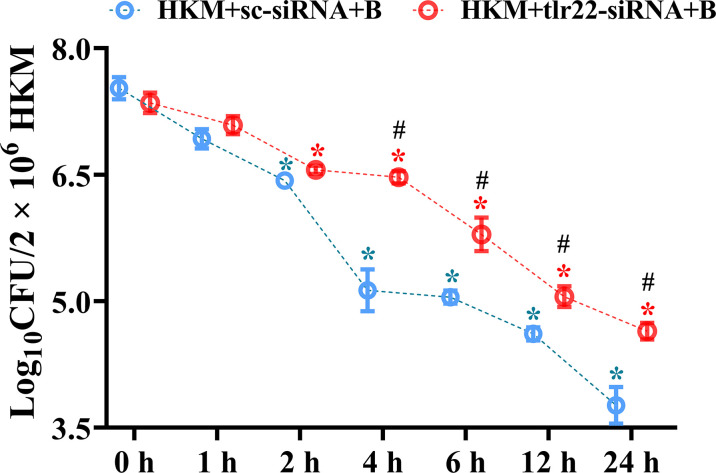
TLR22 signaling restrain *A. hydrophila* replication in HKM. HKM were transfected with sc-siRNA or *tlr22*-siRNA, infected with *A. hydrophila* and bacterial load was enumerated at indicated time points. Data represent mean ± SEM (n=3). Asterisk (*) signifies significant difference as compared to 0 h (*p < 0.05), Hashtag (#) signifies significant difference between the group (^#^*p* < 0.05). HKM+sc-siRNA+B, HKM+tlr22-siRNA+B, sc-siRNA and tlr22-siRNA transfected HKM were infected with *A. hydrophila*.

Macrophages help in counteracting bacterial infections. Towards this direction, HKM were infected with *A. hydrophila* and the bacterial load was recorded at indicated time point p.i. We observed time-dependent reduction in the intracellular bacterial load with significant reduction recorded from 4 h p.i. (**Figure 1**).

**Figure 2:**
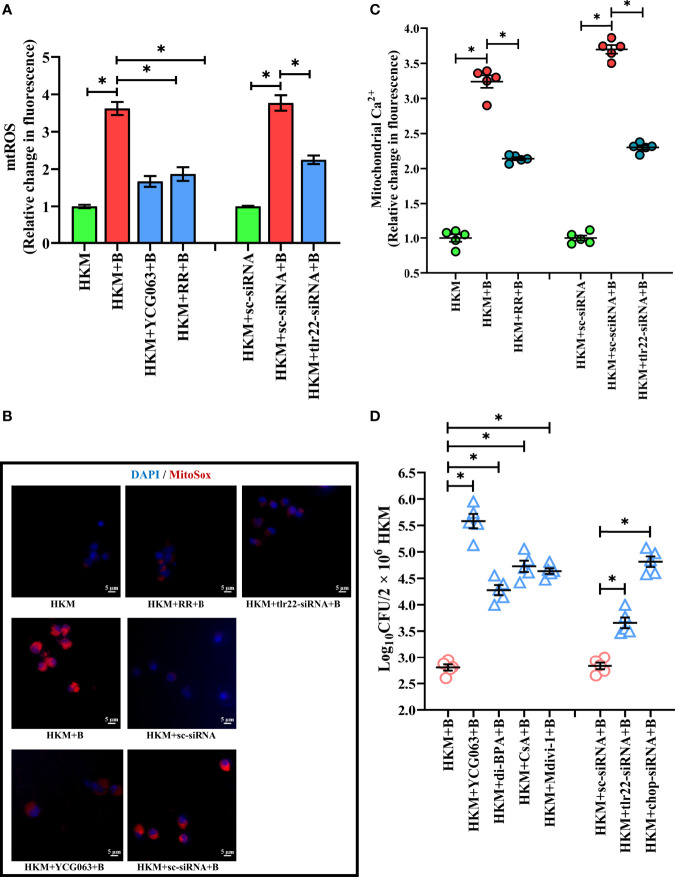
TLR22-induced (Ca^2+^)_mt_ flux instigates pro-apoptotic mtROS generation in *A. hydrophila*-infected HKM. HKM pre-incubated with YCG063, RR or transfected with sc-siRNA, tlr22-siRNA were infected with *A. hydrophila* and at 4 h p.i. **(A)** changes in mtROS levels were measured, and **(B)** changes in mtROS levels were visualized under fluorescence microscope. Vertical bars denote mean ± SEM (n=5). Fluorescence microscopic data is representative of three independent experiments. **(C)** HKM pre-incubated with RR or transfected with sc-siRNA, tlr22-siRNA were infected with *A. hydrophila*, and changes in (Ca^2+^)_mt_ were measured using Rhod-2/AM at 1 h p.i. **(D)** HKM pre-incubated with YCG063, di-BPA, CsA, Mdivi-1 or transfected with sc-siRNA, tlr22-siRNA were infected with *A. hydrophila* and bacterial load were enumerated at 24 h p.i. Data represent mean ± SEM (n=5). Asterisk (*) signifies significant difference between the indicated group (*p < 0.05). HKM, uninfected HKM; HKM+B, HKM infected with *A. hydrophila*; HKM+YCG063+B, HKM+RR+B, HKM+di-BPA, HKM+CsA+B, HKM+Mdivi-1, HKM pre-incubated with YCG063, Ruthenium Red, di-BPA, CsA, Mdivi-1 were infected with *A. hydrophila*; HKM+sc-siRNA, sc-siRNA transfected HKM; HKM+sc-siRNA+B, HKM+tlr22-siRNA+B, sc-siRNA and tlr22-siRNA transfected HKM were infected with *A. hydrophila*.

The next step was to identify the signaling molecules that aid in controlling *A. hydrophila* replication. Our previous studies implicated the role of TLR22 and mtROS in regulating *A. hydrophila* replication (16) and HKM apoptosis (36) respectively. Here we aimed to correlate the two molecular events in *A. hydrophila* pathogenesis. For this, we selected the 4 h time interval because a) significant reduction in intracellular *A. hydrophila* load was observed from 4 h p.i. and b) maximum mtROS production was noted at 4 h p.i. in *A. hydrophila*-infected HKM (36). Thus, HKM transfected with *tlr22*-siRNA were infected with *A. hydrophila* and the changes in mtROS levels were monitored at 4 h p.i. We observed a significant reduction in mtROS production in *tlr22*-knockdown HKM (**Figures 2A, B**) suggesting the role of TLR22 signaling in inducing mtROS production in *A. hydrophila-*infected HKM. Pre-incubation with the mtROS inhibitor (YCG063) attenuated mtROS production in *A. hydrophila-*infected HKM (**Figures 2A, B**). Our results for the first time established the primal role of TLR22 in mtROS production in *A. hydrophila* infection.

Our next step was studying the intermediate molecules that link TLR22 with mtROS generation. Mitochondrial calcium [(Ca^2+^)_mt_] flux plays a major role in mtROS production (37). In line with this, we had reported that *A. hydrophila* infection leads to Ca^2+^ sequestration in mitochondria at 1 h p.i (33). We hypothesized that TLR22 in (Ca^2+^)_mt_ flux thereby compounding *A. hydrophila* pathogenesis. To test this, HKM transfected with *tlr22*-siRNA, were infected with *A. hydrophila* and the (Ca^2+^)_mt_ levels was measured at 1 h p.i., using Rhod-2/AM. The significant reduction in (Ca^2+^)_mt_ levels in *tlr22*-knockdown HKM (**Figure 2C**) suggested that TLR22 signaling positively instigates (Ca^2+^)_mt_ dynamics in *A. hydrophila*-infected HKM. Identifying the molecules that aid in (Ca^2+^)_mt_ flux was the next step and mitochondrial Ca^2+^ uniporter (MCU) was a rational candidate. Impairment of MCU functioning by ruthenium red (RR) attenuated Ca^2+^ influx into the mitochondria (**Figure 2C**) and consequently inhibited mtROS production in *A. hydrophila-*infected HKM (**Figures 2A, B**). Collectively our results suggested that TLR22-induced (Ca^2+^)_mt_ flux through MUP triggers downstream mtROS production in *A. hydrophila* infected HKM.

We followed this by monitoring the effect of *tlr22*-induced mtROS on the intracellular *A. hydrophila* replication. For this, we selected the 24 h time interval and observed that inhibiting the TLR22/mtROS axis significantly increased intracellular *A. hydrophila* load (**Figure 2D**). We had previously reported that the inhibition of mtROS production alleviated HKM apoptosis (36) suggesting *tlr22*-induced mtROS plays anti-bacterial and pro-apoptotic roles in *A. hydrophila* pathogenesis.

### TLR22-mtROS Axis-Induced Hypoxia Instigates Pro-Apoptotic CHOP in *A. hydrophila*-Infected Macrophages

**Figure 3:**
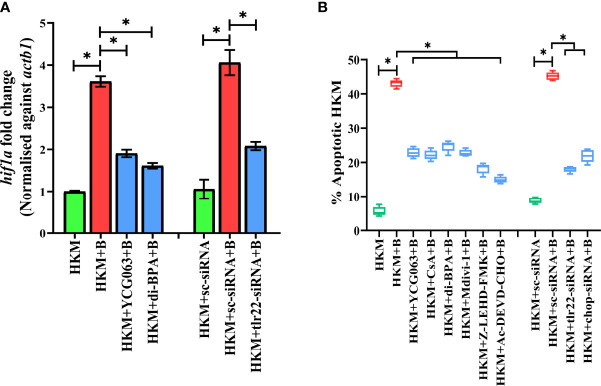
TLR22 induces pro-apoptotic mtROS-dependent HIF-1α activation in *A. hydrophila*-infected HKM. **(A)** HKM pre-incubated with YCG063, di-BPA or transfected with sc-siRNA, tlr22-siRNA were infected with *A. hydrophila*, and the expression of *hif1a* mRNA studied at 4 h p.i. Vertical bars denote mean ± SEM (n=3). Asterisk (*) signifies significant difference between the indicated group (*p < 0.05). **(B)** HKM were pre-incubated with YCG063, CsA, di-BPA, Mdivi-1, Z-LEHD-FMK, Ac-DEVD-CHO or transfected with sc-siRNA, tlr22-siRNA, chop-siRNA were infected with *A. hydrophila* and at 24 h p.i., Hoechst-positive HKM were enumerated at 24 h p.i. Data are presented as box-and-whisker plots (n=5), shows the medians and 25^th^ and 75^th^ percentiles, and the whiskers show 10^th^ and 90^th^ percentiles. HKM, uninfected HKM; HKM+B, HKM infected with *A. hydrophila*; HKM+YCG063+B, HKM+di-BPA+B; HKM pre-incubated with YCG063 and di-BPA respectively were infected with *A. hydrophila*; HKM+sc-siRNA, sc-siRNA transfected HKM; HKM+sc-siRNA+B, HKM+tlr22-siRNA+B, sc-siRNA and tlr22-siRNA transfected HKM respectively were infected with *A. hydrophila*.

Bacterial infection induces hypoxia (17). At the outset, we monitored *hif1a* expression in *A. hydrophila*-infected HKM and observed maximum expression at 6 h p.i. (**Figure S2A**) and selected this time point for subsequent studies. TLR signaling has been implicated in HIF-1α activation (38) and we hypothesized the role of TLR22 in the process. To test this, HKM transfected with *tlr22*-siRNA was infected with *A. hydrophila*, and *hif1a* expression was monitored at 6 h p.i. The significant reduction in *hif1a* expression in *tlr22*-knockdown HKM (**Figure 3A**) suggested the role of TLR22 in inducing hypoxia consequent to *A. hydrophila* infection. HIF-1α inhibitor (dimethyl-BPA) was used as a negative control which effectively repressed *hif1α* expression in the infected HKM (**Figure 3A**). Previous studies have implicated the role of mtROS in hypoxia (39). To study this, HKM pre-incubated with YCG063 were infected with *A. hydrophila* and the *hif1a* expression was monitored at 6 h p.i. Significant reduction in *hif1a* expression confirmed the essential role of mtROS on triggering hypoxia in *A. hydrophila*-infected HKM (**Figure 3A**).

The role of hypoxia in containing bacterial growth is well reported (40). Towards this direction, HKM pre-incubated with dimethyl-BPA were infected with *A. hydrophila*, and bacterial replication was monitored at 24 h p.i. We observed that inhibition of *hif1a* resulted in the significant increase in intracellular *A. hydrophila* (**Figure 2D**). Based on these findings, we suggest that TLR22-induced mtROS triggers hypoxia to counteract intracellular *A. hydrophila* replication.

**Figure 4:**
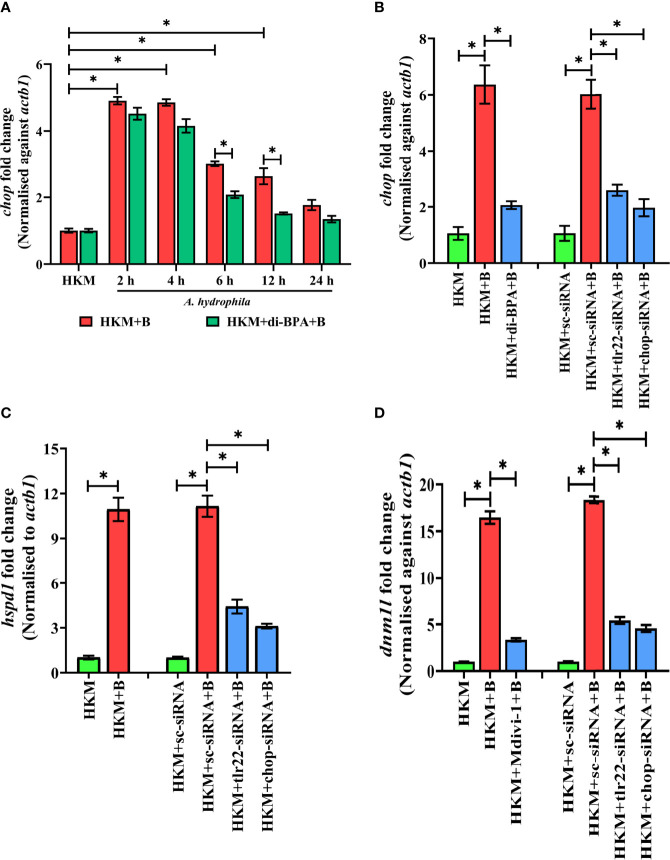
TLR22-induced HIF-1α sustains activation of CHOP triggering UPR^mt^-mediated mitochondrial fragmentation in *A. hydrophila*-infected HKM. **(A)** HKM pre-incubated with di-BPA were infected with *A. hydrophila* and the expression of *chop* mRNA studied at indicated time points. HKM, uninfected HKM; HKM+B, HKM infected with *A. hydrophila*; HKM+di-BPA+B, HKM pre-incubated with di-BPA infected with *A. hydrophila*. **(B)** HKM pre-incubated with di-BPA, Mdivi-1 or transfected with sc-siRNA, tlr22-siRNA, chop-siRNA were infected with *A. hydrophila* and the expression of **(B)** *chop* mRNA studied at 6 h p.i., **(C)** *hspd1* mRNA studied at 6 h p.i., and **(D)** expression of *dnm1l* mRNA studied at 24 h p.i. Vertical bars denote mean ± SEM (n=3). Asterisk (*) signifies significant difference between the indicated group (*p < 0.05). HKM, uninfected HKM; HKM+B; HKM infected with *A. hydrophila*; HKM+di-BPA+B, HKM+Mdivi-1+B, HKM pre-incubated with di-BPA, Mdivi-1 infected with *A. hydrophila*; HKM+sc-siRNA, HKM+sc-siRNA+B, HKM+tlr22-siRNA+B, HKM+chop-siRNA+B, sc-siRNA, tlr22-siRNA, and chop-siRNA transfected HKM were infected with *A. hydrophila*.

Hypoxia triggers apoptosis of macrophages (41). We previously demonstrated the colloquy between CHOP and HKM apoptosis in *A. hydrophila* infection (36). In this line, we presumed the bactericidal role of HIF-1α in *A. hydrophila* infection is attributed to the activation of CHOP. At the outset, we monitored *chop* expression consequent to *A. hydrophila* infection and observed maximum *chop* expression at 2 h p.i. (**Figure 4A**) and thereafter though the levels declined it remained significantly high till 12 h p.i. (**Figure 4A**). To establish the role of hypoxia on CHOP activation, HKM were pre-treated with di-BPA and *chop* expression was monitored at indicated time points p.i. Interestingly, di-BPA pre-incubation had little effect on *chop* expression at early time points (**Figure 4A**) but repressed its expression at later time points i.e., at 6 h and 12 h p.i. (**Figure 4A**) suggesting the regulatory role of HIF-1α on prolonged activation of CHOP in *A. hydrophila*-infected HKM. Also, inhibition of TLR22 signaling attenuated chop expression (**Figure 4B**). Additionally, silencing of *chop* by RNAi led to the significant decline in apoptosis of *A. hydrophila*-infected HKM (**Figure 3B**) together implicating the intermediary role of CHOP in hypoxia-induced apoptosis of *A. hydrophila*-infected HKM.

### CHOP Activates UPR^mt^ in *A. hydrophila-*Infected Macrophages

Excessive mtROS induces proteotoxic stress and in turn, mitochondria trigger mitochondrial-UPR (UPR^mt^) to maintain proteostasis (42, 43). The supra-normal levels of mtROS encouraged us to study UPR^mt^ in *A. hydrophila* pathogenesis. *hspd1* encodes for the mitochondrial chaperone, HSP60 which is a marker for UPR^mt^ (44). At the onset, HKM were infected with *A. hydrophila* and *hspd1* expression monitored at indicated time point p.i. The RT-qPCR data demonstrated maximum fold change in *hspd1* expression at 6 h p.i. and was selected for subsequent studies (**Figure S2B**).

Besides imparting apoptosis, CHOP also plays a role in initiating UPR^mt^ (45). To study the link between *chop* and UPR^mt^ in *A. hydrophila* pathogenesis, HKM were transfected with *chop*-siRNA and *hspd1* expression monitored at 6 h p.i. We noticed a significant reduction in *hspd1* expression in *chop*-knockdown HKM (**Figure 4C**) implicating the role of CHOP in inducing UPR^mt^ in *A. hydrophila* infection.

### UPR^mt^ Induces Mitochondrial Fragmentation in *A. hydrophila*-Infected Macrophages

UPR^mt^ impacts the mitochondrial architecture (46). In absence of prior information, we assessed whether *A. hydrophila* infection alters mitochondrial network, and for that, we monitored the expression of *dnm1l* gene, that encodes for the cytosolic GTPase protein, DRP1 regulating mitochondrial fission (47). We observed a maximum fold increase in *dnm1l* expression at 24 h p.i. (**Figure S3A**) and selected this time point for subsequent studies. Mitochondrial fission was further validated by studying the mitochondrial network architecture. Towards that direction, HKM were infected with *A. hydrophila* then stained with MitoTracker Green dye and visualized at 24 h p.i. by confocal microscopy. Unlike normal mitochondria which form elongated networks; the fragmented mitochondria appear rod-shaped (**Figure S3B**). The presence of rod-like mitochondria confirmed that *A. hydrophila*-induced mitochondrial fission in infected HKM. TLR signaling has been reported in regulating mitochondrial network dynamics (48) and therefore we hypothesized the role of TLR22 in the process. In this line, HKM transfected with *tlr22*-siRNA was infected with *A. hydrophila*, and *dnm1l* mRNA expression was monitored at 24 h p.i. The significant reduction in *dnm1l* mRNA expression in *tlr22*-knockdown HKM (**Figure 4D**) clearly indicates the role of TLR22 as a regulator of mitochondrial network architecture consequent to *A. hydrophila* infection. Together, these results for the first time suggested the occurrence of mitochondrial fragmentation in *A. hydrophila* infection.

Having established that *A. hydrophila* infection induces mitochondrial fragmentation, we asked whether inhibition of UPR^mt^ would reverse the mitochondrial fragmentation. CHOP being a regulator for UPR^mt^ response was selected for the study. Thus, HKM transfected with *chop*-siRNA, were infected with *A. hydrophila* and the expression of *dnm1l* was monitored at 24 h p.i. We observed that the silencing of *chop* resulted in a significant reduction in *dnm1l* expression (**Figure 4D**) which implies that alleviating UPR^mt^ restores mitochondrial network architecture in *A. hydrophila*-infected HKM.

### Mitochondrial Fragmentation Induces Mitochondrial Dysfunction Triggering Caspase-9-Mediated HKM Death

**Figure 5:**
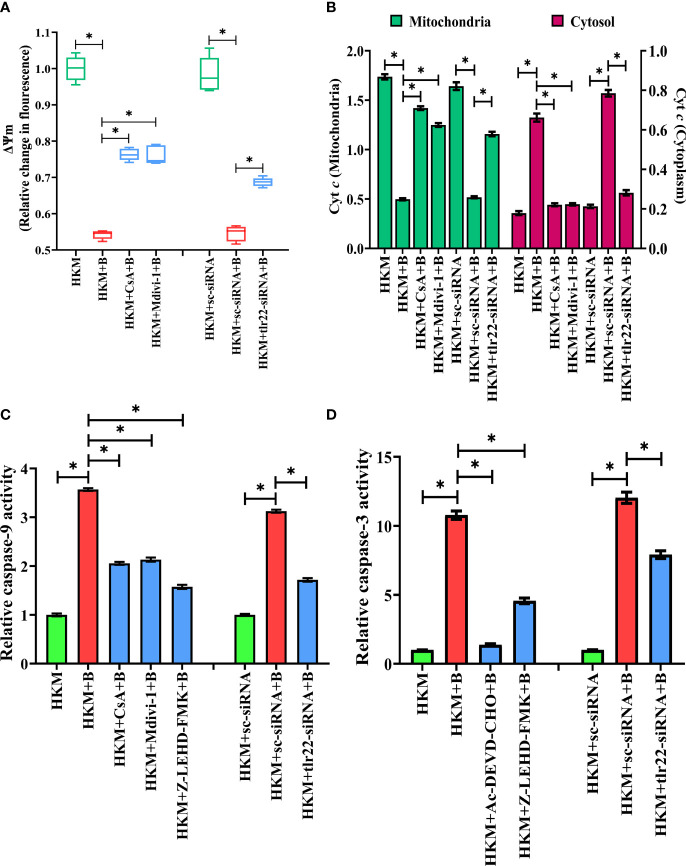
TLR22-induced mitochondrial fragmentation dissipates ψ_m_ and triggers Cyt *c* release actvating caspase-9/caspase-3 axis in *A. hydrophila*-infected HKM. HKM pre-incubated with CsA, Mdivi-1 or transfected with sc-siRNA, tlr22-siRNA were infected with *A. hydrophila* and at 24 h p.i., **(A)** changes in ψ_m_ were studied using Rhodamine-123, and **(B)** changes in Cyt *c* release was studied. Data are presented as box-and-whisker plots (n=5), shows the median and 25^th^ and 75^th^ percentiles, and the whiskers show 10^th^ and 90^th^ percentiles. Vertical bars denote mean ± SEM (n=5). HKM pre-incubated with CsA, Mdivi-1, Z0LEHD-FMK, Ac-DEVD-CHO or transfected with sc-siRNA, tlr22-siRNA were infected with *A. hydrophila* and at 24 h p.i., **(C)** caspase-9 activity and **(D)** caspase-3 activity were studied. Vertical bars denote mean ± SEM (n=3). Asterisk (*) signifies significant difference between the indicated group (*p < 0.05). HKM, uninfected HKM; HKM+B, HKM infected with *A. hydrophila*; HKM+CsA+B, HKM+Mdivi-1 +B, HKM+Z-LEHD-FMK, HKM+Ac-DEVD-CHO; HKM pre-incubated with CsA, Mdivi-1, Z-LEHD-FMK, Ac-DEVD-CHO were infected with *A. hydrophila*; HKM+sc-siRNA, sc-siRNA transfected HKM; HKM+sc-siRNA+B, HKM+tlr22-siRNA+B, HKM+chop-siRNA+B, sc-siRNA, tlr22-siRNA and chop-siRNA transfected HKM were infected with *A. hydrophila*.

Mitochondrial dynamics is crucial in maintaining mitochondrial functioning (49). Depolarization of ΔΨ_m_ and release of pro-apoptotic protein such as Cyt *c* are clear signals of mitochondrial dysfunction (50). To correlate mitochondrial fragmentation with mitochondrial dysfunctioning, HKM were pre-incubated with mitochondrial fission inhibitor, Mdivi-1, then infected with *A. hydrophila* and the changes in ΔΨ_m_ and Cyt *c* release studied at 24 h p.i. We observed that Mdivi-1 pre-incubation attenuated *dnm1l* expression (**Figure 4D**) and ΔΨ_m_ (**Figure 5A**) of *A. hydrophila*-infected HKM. Consequently, Mdivi-1 inhibited Cyt *c* release (**Figure 5B**), repressed the activation of caspase-9/caspase-3 axis (**Figures 5C, D**), HKM apoptosis (**Figure 3B**) and increased the number of intracellular *A. hydrophila* (**Figure 2D**). These results portray that mitochondrial fragmentation triggers caspase-9/caspase-3-mediated apoptosis of *A. hydrophila*-infected HKM thereby aiding the clearance of intracellular bacteria.

We questioned the role of TLR22 in the activation of caspase-9/caspase-3 axis. To study this, HKM were transfected with *tlr22*-siRNA and changes in ΔΨ_m_ and the translocation of Cyt *c* were studied in *A. hydrophila*-infected HKM. We observed that the loss in ΔΨ_m_ and Cyt *c* translocation was repressed in the *tlr22*-knockdown HKM (**Figure 5B**). Additionally, RNAi studies also demonstrated that both caspase-9 (**Figure 5C**) and caspase-3 (**Figure 5D**) activity to be attenuated in *tlr22*-knockdown HKM. CsA (mPTP inhibitor) was used as the control which repressed the activation of caspase-9/caspase-3 axis in *A. hydrophila*-infected HKM (**Figures 5C, D**). Altogether, our results establish the role of TLR22 in the activation of caspase-9/caspase-3 axis in *A. hydrophila*-induced HKM apoptosis.

## Discussion

During bacterial infection, the host faces a unique set of challenges and relies on efficient TLR signaling to counter the pathogens. However, despite the expanding knowledge of TLR signaling, the innate immune axes regulated by TLRs are not well understood, particularly in fish. TLR22 is an important PRR in non-mammalian aquatic vertebrates, including fish, but its role in immunity remains obscure. Studies have highlighted its contradictory role in fish innate immune system. Some report suggest it triggers inflammatory response (51) whereas other report suggest it acts as an equalizer to suppress excessive inflammation (52). This study highlights the role of TLR22 signaling in mounting mitochondria-mediated innate immune responses in *A. hydrophila* pathogenesis in fish.

Recent pieces of evidence have highlighted mitochondria as a central hub in the innate immune signaling pathway (53). Nonetheless, reports concerning its involvement in fish innate immunity are lacking. Mitochondria are major sites for ROS production and our previous studies suggested that *A. hydrophila* induces mtROS production in HKM with pro-apoptotic implications (36). We also reported the pro-apoptotic role of TLR22 in *A. hydrophila* pathogenesis (16), but the possibility that TLR22 signaling regulates mtROS generation has remained unexplored. Towards that end, we observed a significant reduction in mtROS levels in *A. hydrophila*-infected HKM in the absence of TLR22 signaling. This finding for the first time established the role of TLR22 in modulating mtROS production thereby impacting bacterial pathogenesis. Previous studies have documented TLR-dependency of mtROS production consequent to microbial infections (5). With the consensus of previous reports, and our own observations we conclude that the ability to trigger mtROS following pathogenic insult is conserved among the members of the TLR superfamily.

The protective role of mtROS in antimicrobial immune defense is well established in mammals (5, 54). We observed an inverse correlation between mtROS levels and intracellular *A. hydrophila* load and inhibiting mtROS generation increased the bacterial load demonstrating the antimicrobial role of mtROS in fish. To the best of our knowledge, this is the first report on the role of mtROS as a microbicidal factor in fish. Our results are in accord with several previous reports in mammals (5, 54) thereby implicating this event as an evolutionarily conserved innate immune trait. We suggest that TLR22-induced mtROS triggers HKM apoptosis thereby aiding in the removal of infected cells along with pathogens.

The next step was to understand how TLR22 influenced mtROS production in the infected HKM. (Ca^2+^)_mt_ overload is suggested as one of the major causes for enhancing mtROS production under pathophysiological conditions (55). Mitochondria play a paramount role in regulating the spatiotemporal patterns of Ca^2+^ signaling thus controlling cell survival and death (56). It can uptake (Ca^2+^)_C_ through the MCU complex and our previous studies also revealed that *A. hydrophila* triggers (Ca^2+^)_C_ influx through MCU (33, 36). To correlate this with TLR22, we used the RNAi approach and measured (Ca^2+^)_mt_ levels. The marked decline in (Ca^2+^)_mt_ levels in *tlr22*-knockdown HKM implicated TLR22 in initiating (Ca^2+^)_C_ influx inside mitochondria. At present, we do not know how TLR22 influences (Ca^2+^)_mt_ influx. It has been suggested that TLRs *per se* do not modulate MCU activity, they rather control Ca^2+^ mobilization from intracellular stores (57) which subsequently influence MCU activity and prompts (Ca^2+^)_mt_ influx (58). This highlights the possibility that TLR22 might be playing an indirect role in augmenting (Ca^2+^)_mt_ levels in *A. hydrophila*-infected HKM. It has also been observed that following TLR2/4 ligation the mitochondrial adaptor protein ECSIT interacts with TRAF6 to up-regulate mtROS production in macrophages (5). Additionally, the mitochondrial transcription factor A (TFAM) is reported to play a role in triggering mtROS production in response to TLR4 signaling. The conservancy in TLR signaling makes it an interesting proposition to study whether similar cascades of events are initiated following TLR22 activation in *A. hydrophila*-infected HKM.

The link between (Ca^2+^)_C_ influx into mitochondria and mtROS production in *A. hydrophila* pathogenesis has been established (33, 36). RyR and IP_3_R are Ca^2+^ channels, accountable for the efflux of (Ca^2+^)_ER_ to the cytosol (59). Our previous studies suggested that inhibition of IP_3_R and RyR significantly attenuated (Ca^2+^)_mt_ uptake and HKM apoptosis (33, 36). The influx of Ca^2+^ is essential for the normal functioning of mitochondria unless there is an overload that induces structural-functional alterations with pro-apoptotic implications (37). Based on cumulative evidences, we suggest that consequent to *A. hydrophila* infection TLR22 signaling initiates the influx of (Ca^2+^)_C_ released by ER into mitochondria to buffer the (Ca^2+^)_C_ levels and protect the HKM till it reaches a critical threshold whose transgression leads to structural-functional alterations in the organelle. Finding this critical threshold will be important in deciding Ca^2+^ concentration regulated by TLR22 in different sub-cellular compartments for triggering apoptosis in bacterial infection.

We were intrigued by how mtROS signals HKM apoptosis. Bacterial infections trigger hypoxia-induced host cell apoptosis and HIF-1α plays an important role in the process (17, 40). We hypothesized the role of mtROS in HIF-1α expression. Towards this direction, we recorded significant upregulation in *hif1a* expression in *A. hydrophila*-infected HKM. Using pharmacological inhibitors and RNAi studies, we further noted that inhibiting TLR22 signaling or mtROS generation attenuated *hif1a* expression indicating the role of TLR22/mtROS axis in HIF-1α activation in *A. hydrophila*-infected HKM. Our results are in consonance with previous reports which suggested mtROS is an essential intermediate in HIF-1α activation and TLR signaling plays a primal role in initiating the chain of events (39, 60).

The next step was to study the role of HIF-1α in regulating *A. hydrophila* pathogenesis. The significant reduction in HKM apoptosis and concomitant increase in bacterial burden implicated the anti-bacterial role of HIF-1α in *A. hydrophila* infection. Various reports are in line with our observations which suggested hosts deficient in HIF-1α are susceptible to bacterial infection (18, 40). The mechanism underlying HIF-1α signaling in fish is not well understood. CHOP has been implicated in HIF-1α signaling (61). Previously, we had also reported the role of CHOP in the apoptosis of *A. hydrophila*-infected HKM (36). At the outset, we questioned whether TLR22 influences CHOP expression, and our RNAi results clearly suggested CHOP activation downstream of TLR22 signaling. Our next step was establishing the link between HIF-1α and CHOP. We observed that inhibition of HIF-1α interfered with prolonged expression of *chop* implicating the role of HIF-1α in sustaining CHOP activation in *A. hydrophila* infection. These results suggested that CHOP is an intermediary molecule in the pro-apoptotic TLR22/HIF-1α axis in *A. hydrophila* pathogenesis.

Exposure to pathogens causes mitochondrial dysfunction and UPR^mt^ activation (28) and to the best of our knowledge, there are no such precedents in *A. hydrophila* pathogenesis. Importantly, UPR^mt^ has also not been reported in fish. Hence, our first aim was to study UPR^mt^ in *A. hydrophila*-infected HKM. Hspd1 is considered as a marker for UPR^mt^ (62, 63) and we noticed TLR22-mediated *hspd1* expression in *A. hydrophila* infection. Our findings assume significant importance because a) it is the first report on UPR^mt^ in fish and b) reflects the involvement of TLR22 signaling in UPR^mt^ thereby impacting microbial pathogenesis. Next, we investigated how UPR^mt^ is regulated by TLR22. CHOP playing a key role in UPR^mt^ activation (46) was our prime target. We did observe that CHOP silencing attenuated UPR^mt^ activation in the infected HKM. CHOP binding sites have been reported in UPR^mt^ elements triggering their transcriptional activation (63). To this, we concluded that consequent to *A. hydrophila* infection TLR22 signaling converges at HIF-1α which in turn induced CHOP-mediated activation of UPR^mt^ in infected HKM. Future studies aimed at identifying other molecules that influence UPR^mt^ activation will help in understanding the role of mitochondrial proteostasis in regulating microbial pathogenesis in fish.

Next, we questioned the implications of the UPR^mt^ activation in *A. hydrophila* pathogenesis. Activation of UPR^mt^ is a primary retort to defend mitochondrial homeostasis (42) but prolonged UPR^mt^ impacts mitochondrial homeostasis triggering cell death (64). Drp1 plays a critical role in mitochondrial fission (23) and we observed significant upregulation in *dnm1l* expression and fragmented mitochondrial network in the *A. hydrophila*-infected HKM. Based on these findings, we suggest that *A. hydrophila* infection affects mitochondrial homeostasis by favoring mitochondrial fission. This finding is in line with previous reports documenting bacteria-induced mitochondrial fission in mammalian cells (65). Various studies have reported TLR-mediated switching of mitochondrial morphology to fission from fusion (48). We observed that silencing of TLR22 attenuated the expression of *dnm1l* suggesting TLR22 functions as mitochondrial-fission regulator in *A. hydrophila* pathogenesis.

Though the biological importance of mitochondrial fission is unclear, reports suggested it is a prerequisite in screening the damaged mitochondria for mitophagic culling (66). Previously, we had reported failure of the autophagic machinery in *A. hydrophila* infection leading to accretion of damaged mitochondrial fragments (36). This prompted us to investigate the consequences of mitochondrial fragmentation in *A. hydrophila*-infected HKM. Mitochondrial fission leads to a loss in Δψ_m_ and the opening of mPTP thereby releasing pro-apoptotic cyt *c* (67, 68). We observed that inhibiting TLR22 signaling and mitochondrial fission reinstated Δψ_m_ and inhibited cyt *c* release thereby attenuating HKM apoptosis. These results are in consonance with a previous report documenting mitochondrial fission triggers cyt *c* release (67). Additionally, the intracellular survival of *A. hydrophila* was also significantly increased on inhibiting mitochondrial fission, highlighting the relevance of mitochondrial network homeostasis in *A. hydrophila* pathogenesis. Similar reports support our finding which suggested that mitochondrial fission directs cells towards apoptosis and regulates bacterial pathogenesis (69, 70).

Cyt *c* is a central molecule in the activation of caspase-9/caspase-3 axis (71). Our earlier reports demonstrated the activation of caspase-9 consequent to *A. hydrophila* infection (33). Here we aimed to establish the link between TLR22 and caspase-9/caspase-3 axis. We noted that the caspase-9/caspase-3 axis was repressed in *tlr22*-knockdown HKM suggesting TLR22 signaling culminates in caspase-9/caspase-3-mediated apoptosis of *A. hydrophila-*infected HKM. Collectively, our findings implicate the pro-apoptotic role of mitochondrial network dynamics in TLR22-mediated apoptosis of *A. hydrophila*-infected HKM.

**Figure 6:**
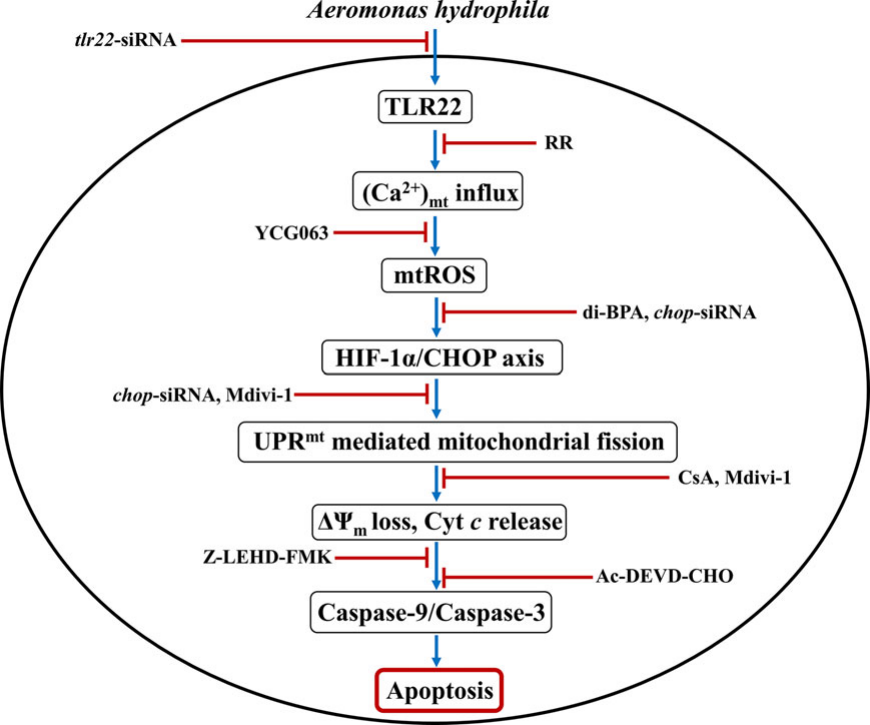
Overview of the study. TLR22-induced Ca^2+^ influx into mitochondria triggers mtROS generation leading to downstream activation of HIF-1α/CHOP axis. Activated CHOP instigates UPR^mt^-mediated fragmentation of the mitochondrial network leading to mitochondrial dysfunction which activates caspase-9/caspase-3 axis-mediated apoptosis of *A. hydrophila*-infected HKM.

To conclude, our findings established the primal role of TLR22 in utilizing mitochondria-derived immune signaling cues integral in controlling the onset and pathogenesis of *A. hydrophila* infection. We propose that TLR22 serves as a conduit linking *A. hydrophila* infection with the influx of (Ca^2+^)_C_ into the mitochondria, entailing mtROS production. Enhanced mtROS induces HIF-1α favoring the sustained activation of CHOP which triggers UPR^mt^-induced mitochondrial fission and organelle dysfunctioning. Compromised autophagy leads to the accumulation of dysfunctional mitochondria which releases Cyt *c* triggering downstream caspase-9/caspase-3-mediated HKM apoptosis and the clearance of *A. hydrophila* (**Figure 6**). Thus, it will be exciting to extrapolate this current understanding and investigate how innate immune pathways administrated by TLR22 and mitochondria can be translated into active therapeutics to boost the immune system of fish against *A. hydrophila* and control motile aeromonad septicaemia and ulcerative diseases.

## Data Availability Statement

The datasets presented in this study can be found in online repositories. The names of the repository/repositories and accession number(s) can be found below: https://www.ncbi.nlm.nih.gov/, MZ882392.

## Ethics Statement

The studies were carried out according to the guidelines issued by the Committee for the Purpose of Control and Supervision of Experiments on Animals (CPCSEA), Govt. of India, and permitted by the Animal Ethics Committee (DU/ZOOL/IAEC-R/2013/33), University of Delhi.

## Author Contributions

MK: Conceptualization, Investigation, Methodology, Validation, Visualization, Writing – original draft, Writing – review and editing, Formal analysis. SS: Investigation, Methodology, Writing – review and editing. JK: Investigation, Writing – review and editing. MH: Methodology. UH: Methodology. SM: Conceptualization, Resources, Supervision, Writing – original draft, Writing – review and editing. All authors contributed to the article and approved the submitted version.

## Funding

MK, SS, and JK were supported by U.G.C.-NET fellowship (Government of India). MH and UH were supported by fellowship provided by South Asian University, India.

## Conflict of Interest

The authors declare that the research was conducted in the absence of any commercial or financial relationships that could be construed as a potential conflict of interest.

## Publisher’s Note

All claims expressed in this article are solely those of the authors and do not necessarily represent those of their affiliated organizations, or those of the publisher, the editors and the reviewers. Any product that may be evaluated in this article, or claim that may be made by its manufacturer, is not guaranteed or endorsed by the publisher.

## Supplementary Material

Supplementary Figure 1 Transfection of tlr22-siRNA and chop-siRNA attenuates tlr22 and chop gene expression in HKM. HKM transfected with sc-siRNA, tlr22-siRNA or chop-siRNA and tlr22, chop expression was studied using RT-qPCR. Vertical bars represent mean ± S.E (n=3). Asterisk (*) signifies significant difference between indicated groups (**p*<0.05). HKM+sc-siRNA, HKM transfected with sc-siRNA; HKM+tlr22-siRNA, HKM transfected with tlr22-siRNA; HKM+chop-siRNA, HKM transfected with chop-siRNA.

Supplementary Figure 2 *A. hydrophila* infection triggers *hif1a* and *hspd1* mRNA expression in HKM. HKM were infected with *A. hydrophila* and at indicated time points **(A)** *hif1a* mRNA expression, and **(B)** *hspd1* mRNA expression were studied. Vertical bars denote mean ± SEM (n=3). Asterisk (*) signifies significant difference between the indicated group (**p*<0.05). HKM, uninfected HKM; HKM+B, HKM infected with *A. hydrophila*.

Supplementary Figure 3 *A. hydrophila* induces mitochondrial fragmentation in infected HKM. HKM were infected with *A. hydrophila* and at indicated time points **(A)** *dnm1l* mRNA expression was studied. **(B)** HKM were infected with *A. hydrophila* and morphology of mitochondrial network studied at 24 h p.i. HKM were washed, stained with MitoTracker green and DAPI, mounted and visualized under microscope (Scale – 2 µm).
