# Supplementary figures and images for: TLR22-Induced Pro-Apoptotic mtROS Abets UPRmt-Mediated Mitochondrial Fission in Aeromonas hydrophila-Infected Headkidney Macrophages of Clarias gariepinus
Source: Front Immunol. 2022 Jul 4;13:931021. doi: 10.3389/fimmu.2022.931021 (PMC9292580; doi:10.3389/fimmu.2022.931021)

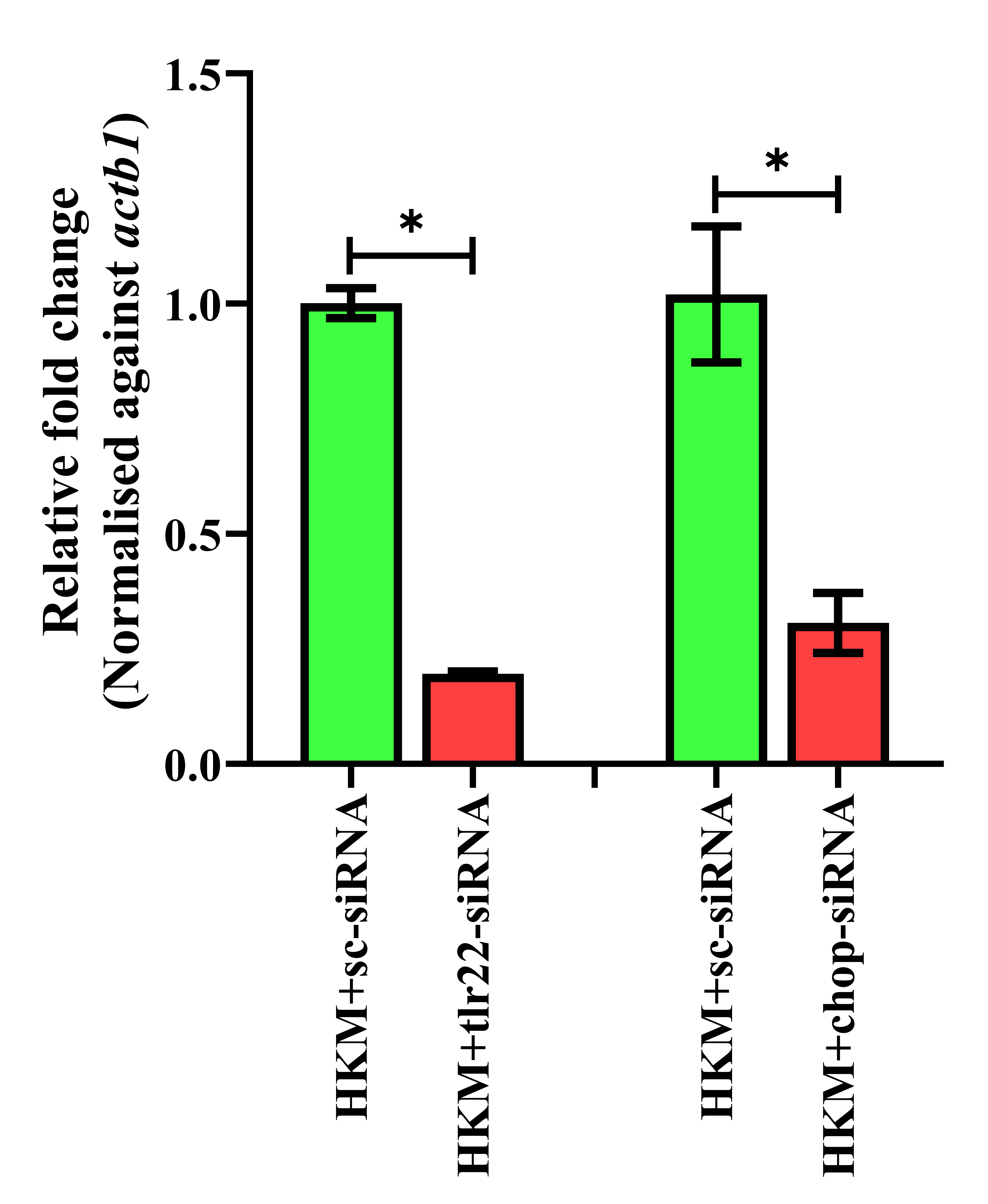

Supplement: Supplementary Figure 1 — Transfection of tlr22-siRNA and chop-siRNA attenuates tlr22 and chop gene expression in HKM. HKM transfected with sc-siRNA, tlr22-siRNA or chop-siRNA and tlr22, chop expression was studied using RT-qPCR. Vertical bars represent mean ± S.E (n=3). Asterisk (*) signifies significant difference between indicated groups (*p<0.05). HKM+sc-siRNA, HKM transfected with sc-siRNA; HKM+tlr22-siRNA, HKM transfected with tlr22-siRNA; HKM+chop-siRNA, HKM transfected with chop-siRNA. [file Image_1.tif]

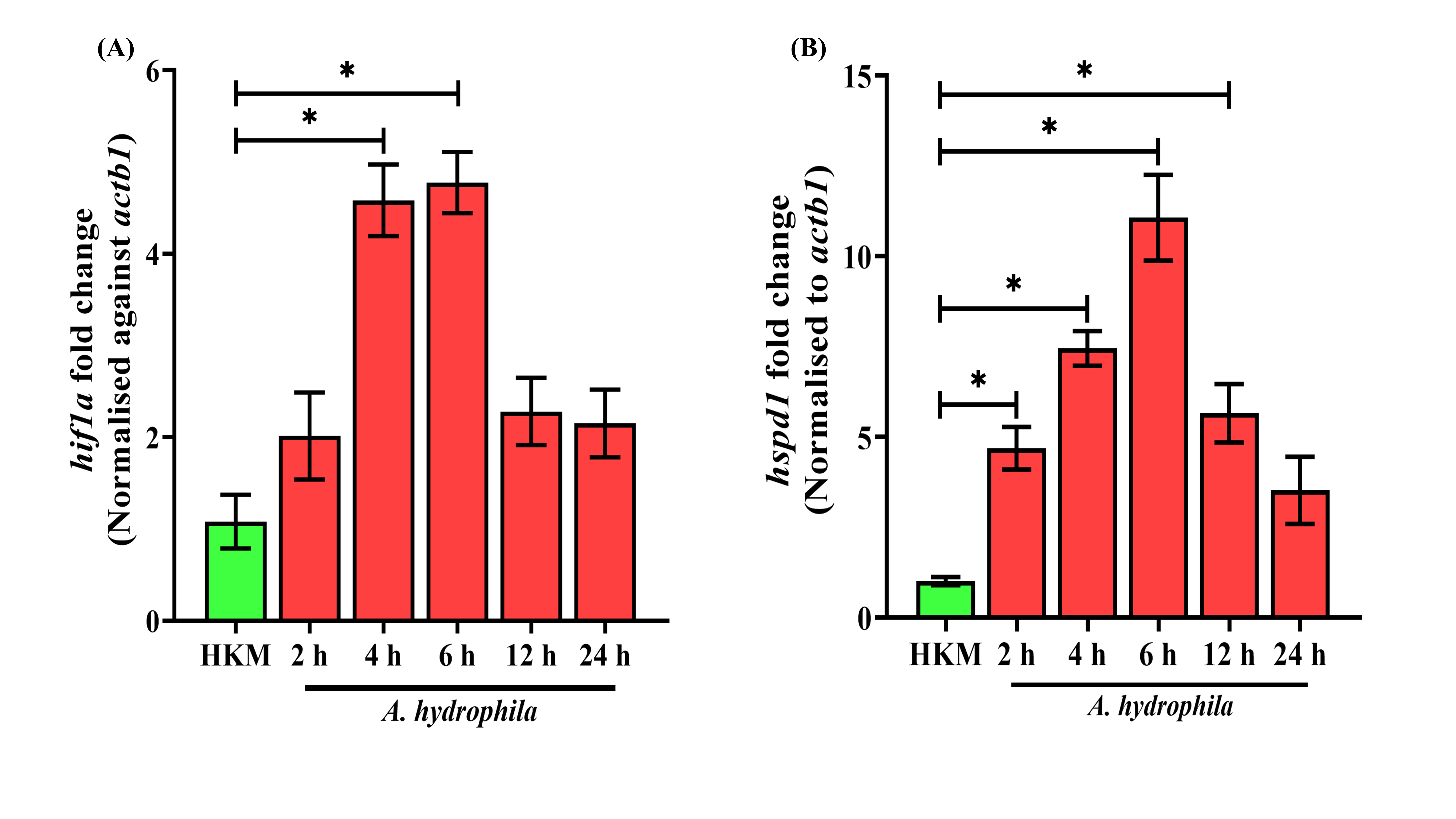

Supplement: Supplementary Figure 2 — A. hydrophila infection triggers hif1a and hspd1 mRNA expression in HKM. HKM were infected with A. hydrophila and at indicated time points (A) hif1a mRNA expression, and (B) hspd1 mRNA expression were studied. Vertical bars denote mean ± SEM (n=3). Asterisk (*) signifies significant difference between the indicated group (*p<0.05). HKM, uninfected HKM; HKM+B, HKM infected with A. hydrophila. [file Image_2.tif]

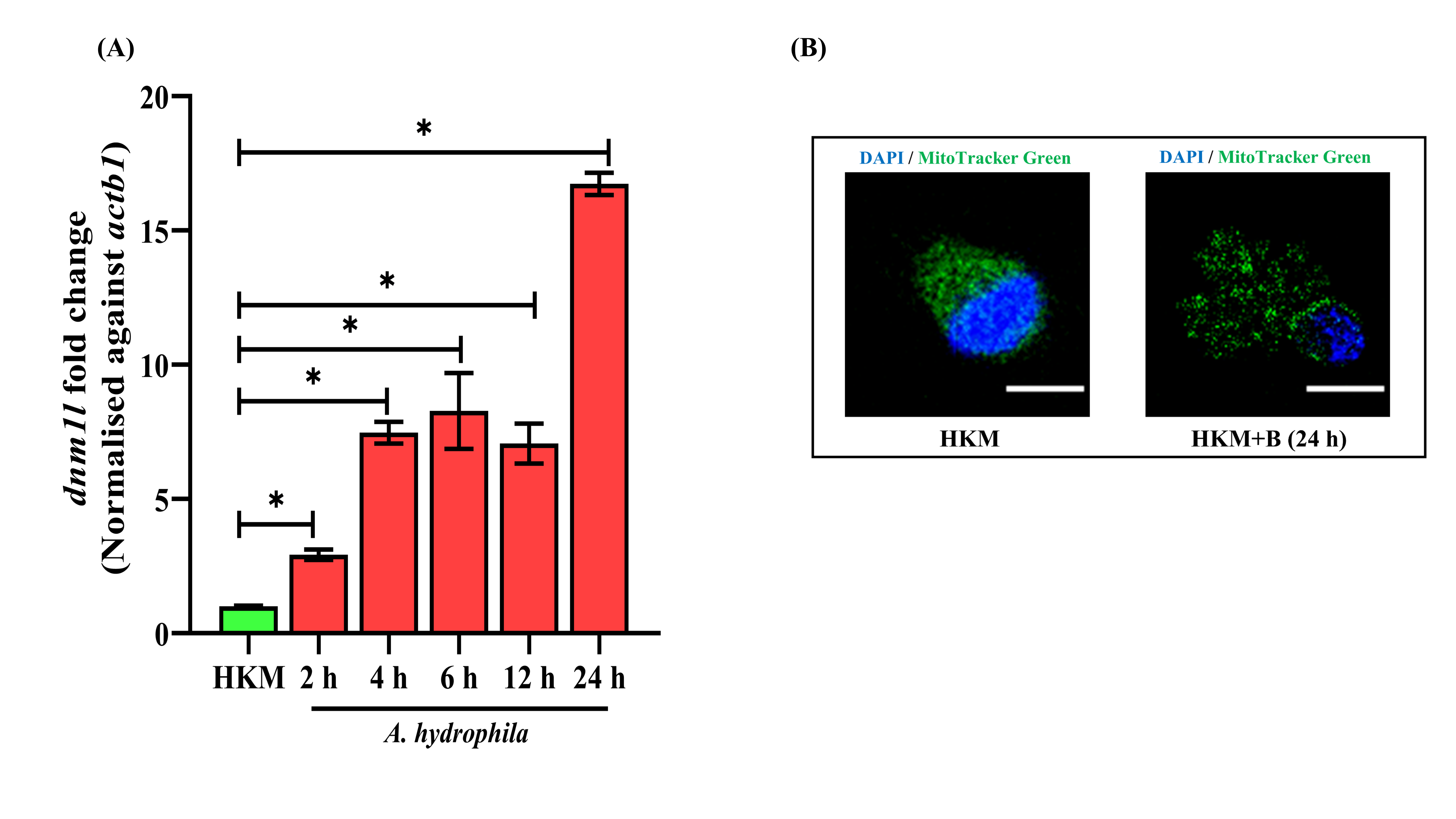

Supplement: Supplementary Figure 3 — A. hydrophila induces mitochondrial fragmentation in infected HKM. HKM were infected with A. hydrophila and at indicated time points (A) dnm1l mRNA expression was studied. (B) HKM were infected with A. hydrophila and morphology of mitochondrial network studied at 24 h p.i. HKM were washed, stained with MitoTracker green and DAPI, mounted and visualized under microscope (Scale – 2 µm). [file Image_3.tif]
