# Supplementary material for: TLR22-Induced Pro-Apoptotic mtROS Abets UPRmt-Mediated Mitochondrial Fission in Aeromonas hydrophila-Infected Headkidney Macrophages of Clarias gariepinus
Source: Front Immunol. 2022 Jul 4;13:931021. doi: 10.3389/fimmu.2022.931021 (PMC9292580; doi:10.3389/fimmu.2022.931021)
Supplement: Supplementary file 4 [file Table_1.docx]

| ***dnm1l* gene sequence (Acc. No. – MZ882392)** |
| --- |
| ATGGAGGCTCTTATTCCTGTCATTAACAAGCTGCAGGATGTGTTTAACACCGTTGGAGCGGATATCATCCAGCTGCCGCAGATTGCTGTGGTCGGGACTCAGAGCAGTGGGAAAAGCTCTGTGTTGGAGAGTCTGGTTGGCAGAGACCTCTTGCCTCGCGGTACTGGCATTGTGACGCGCAGACCACTCATCCTACAGCTGGTGCACGTGGACCCAGAGGACCGGAGAAAGACGAGTGAGGAGAACGGTGTAGAGGGTGAAGAATGGGGTAAATTTCTACATACCAAAAATAAGATCTATACAGATTTTGACGAAATCAGGCAAGAAATTGAATCTGAAACGGAACGAATTTCTGGCAACAACAAGGGCATCAGCAGTGAGCCAATTCACCTAAAGATCTTTTCTCCTCATGTGGTAAACCTCACGTTGGTGGATCTGCCTGGCATTACAAAGGTTCCTGTTGGGGACCAACCCAAAGATATTGAGGTCCAAATTCGTGAGCTGATCCTCCAGTACATTAGCAACCCAAACTGCATTATTTTGGCTGTTACAGCCGCCAACACAGACATGGCTACGTCCGAGGCTCTTAAAGTGGCTCGTGAGGTCGACCCTGATGGCCGAAGGACACTAGCTGTGGTGACAAAACTGGATCTGATGGACGCTGGCACAGACGCCATGGACGTACTCATGGGCAGAGTCATTCCTGTTAAACTGGGACTCATAGGAGTCGTCAATAGGAGTCAACTTGATATCAACAACAAGAAATCAGTGGCTGATGCAATCCGTGACGAGTATGCTTTCCTCCAGAAGAAGTATCCTTCCTTAGCTAACAGAAACGGAACCAAGTACCTGGCCAGAACGTTGAATAGGTTGCTGATGCACCACATTCGGGATTGTCTGCCAGAGCTAAAGACACGTATCAACGTCCTTGCCGCTCAGTACCAGTCTCTGCTCAGCAGCTACGGAGAGCCTGTGGAGGACAAGAGCGCCACCCTGCTGCAGCTCATCACCAAGTTCGCCGCAGAGTACTGCAACACTAT |
